# Circulating *PTGS2*, *JAG1*, *GUCY2C* and *PGF* mRNA in Peripheral Blood and Serum as Potential Biomarkers for Patients with Metastatic Colon Cancer

**DOI:** 10.3390/jcm10112248

**Published:** 2021-05-22

**Authors:** Cristina Jimenez-Luna, Encarnación González-Flores, Raul Ortiz, Luis J. Martínez-González, Alba Antúnez-Rodríguez, Manuela Expósito-Ruiz, Consolación Melguizo, Octavio Caba, Jose Prados

**Affiliations:** 1 Institute of Biopathology and Regenerative Medicine (IBIMER), Center of Biomedical Research (CIBM), University of Granada, 18100 Granada, Spain; crisjilu@ugr.es (C.J.-L.); roquesa@ugr.es (R.O.); ocaba@ugr.es (O.C.); jcprados@ugr.es (J.P.); 2 Department of Anatomy and Embryology, Faculty of Medicine, University of Granada, 18071 Granada, Spain; 3 Instituto de Investigación Biosanitaria ibs. Granada, 18012 Granada, Spain; encarnagonzalezflores@gmail.com; 4 Medical Oncology Service, Hospital Virgen de las Nieves, 18014 Granada, Spain; 5 GENyO, Centre for Genomics and Oncological Research, Pfizer-University of Granada-Andalusian Regional Government, 18016 Granada, Spain; luisjavier.martinez@genyo.es (L.J.M.-G.); alba.antunez@genyo.es (A.A.-R.); 6 Unit of Biostatistics, Department of Statistics and Operations Research, School of Medicine, University of Granada, 18071 Granada, Spain; mexpositoruiz@ugr.es

**Keywords:** metastatic colon cancer, biomarkers, angiogenesis, liquid biopsy, circulating mRNA, digital PCR

## Abstract

Genes involved in the angiogenic process have been proposed for the diagnosis and therapeutic response of metastatic colorectal cancer (CRC). This study aimed to investigate the value of *PTGS2*, *JAG1*, *GUCY2C* and *PGF*-circulating RNA as biomarkers in metastatic CRC. Blood cells and serum mRNA from 59 patients with metastatic CRC and 47 healthy controls were analyzed by digital PCR. The area under the receiver operating characteristic curve (AUC) was used to estimate the diagnostic value of each mRNA alone or mRNA combinations. A significant upregulation of the *JAG1*, *PTGS2* and *GUCY2C* genes in blood cells and serum samples from metastatic CRC patients was detected. Circulating mRNA levels in the serum of all genes were significantly more abundant than in blood. The highest discrimination ability between metastatic CRC patients and healthy donors was obtained with *PTGS2* (AUC of 0.984) and *GUCY2C* (AUC of 0.896) in serum samples. Biomarker combinations did not improve the discriminatory capacity of biomarkers separately. Analyzed biomarkers showed no correlation with overall survival or progression-free survival, but *GUCY2C* and *GUCY2C*/*PTGS2* expression in serum correlated significantly with the response to antiangiogenic agents. These findings demonstrate that assessment of genes involved in the angiogenic process may be a potential non-invasive diagnostic tool for metastatic CRC and its response to antiangiogenic therapy.

## 1. Introduction

Colorectal cancer (CRC) is the second leading cause of cancer-related death worldwide with an incidence that is on track to increase from 1.8 million new cases in 2018 to 2.5 million in 2035. Despite numerous screening programs for early detection, approximately 20–25% of patients exhibit metastatic disease at the disease onset and 50% of patients eventually develop metastases [1,2,3]. The prognosis of metastatic CRC has improved in recent decades with the use of new treatment strategies, new biological agents and therapy optimization based on the genomic characteristics of the tumor. In fact, *RAS*, *BRAF* and microsatellite instability (MSI) determination as well as some clinical biomarkers such as primary tumor location are essential to properly select patients who are candidates for biological treatments. In spite of these advances, the 5-year overall survival of these patients with advanced disease is still less than 15% [4,5]. Currently, the European Society for Medical Oncology (ESMO) guidelines and the Pan-Asia adaptation recommend chemotherapy (CT) based on doublet cytotoxic combinations of fluorouracil, leucovorin and irinotecan (FOLFIRI) and 5-FU, leucovorin and oxaliplatin (FOLFOX) for metastatic CRC patients. Furthermore, targeted agents, such as EGFR antibody therapy in *RAS* will-type tumors and bevacizumab, are indicated in the first-line treatment of most patients [6,7,8]. In this context, the determination of a metastatic stage in CRC has become a priority in order to implement the most appropriate treatment that can improve the patient’s prognosis.

Tissue biopsy biomarkers have been widely used to predict treatment response and prognosis of tumors. However, the invasiveness of the procedure and their low specificity has led to the use of peripheral blood to easily and non-invasively detect novel biomarkers with the potential to reflect tumor status [9,10,11]. Liquid biopsy has emerged as a promising tool for the follow-up of cancer patients [12]. In CRC, liquid biopsies showed promising clinical utility for early detection [13,14,15], relapse [16], prognostic markers [17], response to therapy [18] and survival [19]. Recently, long non-coding RNAs (lncRNAs) circulating in peripheral blood were proposed as a minimally invasive test for CRC diagnosis [20] and circulating tumor DNA (ctDNA)-based liquid biopsy for RAS mutations has also been used for some CRC diagnostic applications [21]. To this end, new biomarkers in blood directly related to cancer spread are of particular importance.

An essential phenomenon directly related to the development, proliferation and metastasis of CRC is angiogenesis, as demonstrated by four US Food and Drug Administration-approved antiangiogenic agents for metastatic CRC (bevacizumab, ramucirumab, aflibercept and regorafenib) [22]. Among them, bevacizumab, a monoclonal antibody targeted against vascular endothelial growth factor (VEGF), has become a first-line treatment option in combination with chemotherapy for patients with metastatic CRC [5,7,23], improving their response rate and survival [24,25]. Nevertheless, the high expectations raised by preclinical studies using antiangiogenic therapies were not fulfilled in clinical practice due to acquired or primary resistance [26,27,28]. A number of genes involved in the angiogenic process have been identified to be overexpressed in CRC progression and metastatization, and several non-invasive biomarkers have been proposed as potential biomarkers for predicting the prognosis and response to antiangiogenic therapy in this disease [29,30]. Biomarkers such as angiopoietin-2, soluble CD73, human epidermal growth factor receptor 3, hepatocyte growth factor, interleukin-6, stromal cell-derived factor 1 and vascular endothelial growth factor-D (VEGF-D) were tested in relation to antiangiogenic treatment. In fact, low VEGF-D expression has been found to improve bevacizumab response in CRC patients [31,32]. However, most of these studies require validation in larger patient cohorts to know who might benefit from these therapies [33].

Crosstalk between VEGF and other signaling pathways importantly contributes to tumor angiogenesis regulation, through the activation of alternative VEGF-dependent and VEGF-independent pathways [34]. Guanylyl cyclase C (GUCY2C), a receptor member of the family of guanyl cyclases, plays an important role in regulating intracellular cGMP levels, electrolyte homeostasis and cell proliferation in the intestine [35]. Deregulation of cGMP signaling observed in CRC involves the overexpression of GUCY2C and decreased levels of its ligands, resulting in hypofunction of the receptor, which could also contribute to loss of genomic integrity [36]. In last years, prostaglandin-endoperoxide synthase 2 (PTGS2) (also known as COX2), an inducible enzyme involved in the synthesis of prostaglandins that contributes to inflammation, angiogenesis, immune evasion and therapy resistance, has also been widely investigated in the tumor context [37]. Its overexpression has been associated with metastasis and poor prognosis in CRC patients [38]. On the other hand, the VEGF and Notch signaling pathways are two pivotal mechanisms in tumor angiogenesis [34]. In fact, placenta growth factor (PGF) is a ligand of the VEGF family that induces angiogenesis by both VEGF-independent and VEGF-dependent ways [39]. Interestingly, increased PGF levels in tumors resistant to anti-VEGF treatment suggest a possible compensatory role of PGF in angiogenesis induction [40]. In addition, Jagged-1 (JAG1) is a canonical ligand that activates Notch signaling that has been reported to be strongly upregulated in different cancers, including CRC, promoting tumor progression, angiogenesis and recurrence [41,42]. Finally, matrix metalloproteinase-7 (MMP7), a member of the zinc-dependent proteolytic enzymes family, degrades the extracellular matrix favoring tumor invasion, metastasis and angiogenesis by matrix-bound VEGF releasing [43]. Increased levels of this enzyme have been related with poor prognosis in advanced CRC [44].

The aim of this study was to assess the utility of five angiogenesis-related genes as biomarkers for predicting prognosis and response to different chemotherapy regimens combined with bevacizumab. Therefore, on the basis of the results previously reported in the literature and the potential clinical utility of using a panel of biomarkers in order to capture as much biological information as possible, we selected *GUCY2C*, *JAG1*, *PTGS2*, *PGF* and *MMP7* genes and determined circulating RNA levels in both peripheral blood mononuclear cells (PBMCs) and serum from patients with metastatic CRC using digital PCR (dPCR) technology. We demonstrated that *PTGS2*, *GUCY2C* and *JAG1* in serum samples showed a high discrimination ability. Only JAG1 in blood showed a similar accuracy. Different clusters of biomarkers did not significantly improve this discriminatory capacity. However, *GUCY2C* and *GUCY2C*/*PTGS2* in serum also correlated significantly with therapeutic response, although they did not correlate with overall survival or progression-free survival.

## 2. Materials and Methods

### 2.1. Patients’ Characteristics

The clinical parameters of metastatic CRC patients who were finally included in the study after RNA extraction from blood and serum (*n* = 59) are summarized in Table 1. At the time of biomarker sampling, all patients had at least one radiologically visible metastasis. Of the 59 patients, 33 (55.93%) were males and 26 (44.07%) were females. The mean age was 60.12 ± 11.16 years. Two control groups were used for both blood (*n* = 28; 14 (50%) males and 14 (50%) females; mean age, 57.77 ± 5.48 years) and serum analyses (*n* = 19; 14 (73.68%) males and 5 (26.32%) females; mean age, 65.50 ± 6.83 years). The RAS gene was shown to be mutated in 31 (52.54%) patients and 35 (59.32%) patients presented metastasis in more than 1 organ. Regarding therapy, 35 (59.32%) patients received chemotherapy involving antiangiogenic treatment while 24 (40.68%) were not administered any antiangiogenic drug.

### 2.2. Biomarker Correlation with Overall Survival or Progression-Free Survival

A total of 66 patients with CRC were initially recruited and whole blood samples were collected before receiving any anticancer treatment (chemotherapy +/− targeted agents) at the Medical Oncology Service of the Virgen de las Nieves University Hospital (Granada, Spain). All patients were confirmed as metastatic CRC (stage IV). The diagnosis of CRC, which was histologically confirmed by surgery or biopsy and metastatic CRC, was based on imaging studies. In addition, samples from 47 age- and sex-matched healthy controls were selected so that none of them presented any type of tumor or inflammatory pathology and were then obtained from the Andalusian Health System Biobank (Granada, Spain). Assessment of therapeutic response was based on responders/non-responders. The study was approved by the Biomedical Research Ethics Committee of the Andalusian Public Health System in Granada (protocol code PI19/01478; No. 2020522131049; 29 July 2020) and conducted in accordance with the Declaration of Helsinki, and written informed consent was obtained from all participants.

### 2.3. RNA Isolation from Blood Cells and Serum Samples

To extract RNA from blood cells, whole blood samples (6 mL) from each patient were collected into Tempus Blood RNA tubes (Thermo Fisher Scientific, Waltham, MA, USA; CAT: 4342792) and vigorously mixed for at least 10 s after collection. Samples were incubated at room temperature for 24 h and then total RNA from the lysed blood cells was isolated using the Tempus Spin RNA Isolation Reagent Kit (Thermo Fisher Scientific, Waltham, MA, USA; REF: 4378926) following the protocol provided by the manufacturer.

In addition, to obtain serum-circulating RNA, matched peripheral blood samples (8.5 mL) from each patient were collected in BD Vacutainer SSTII advance tubes (Becton Dickinson, Franklin Lakes, NJ, USA) and were allowed to clot for at least 30 min. The serum fraction was obtained by centrifugation at 1400× *g* for 10 min and then stored at −80 °C until processing. For total RNA isolation, 300 µL of serum was concentrated to 200 µL using a vacuum concentrator (Vacufuge plus Vacuum Concentrator, Eppendorf AG, Hamburg, Germany) at 4 °C. Circulating free total RNA was purified with the miRNeasy Serum/Plasma Kit (Qiagen) according to the manufacturer’s instructions. Whole blood and serum samples from healthy controls were collected and processed identically to those from CRC patients. The concentration and quality of purified RNA were assayed using a NanoDrop 2000c spectrophotometer (Thermo Fisher Scientific) and a 2100 Bioanalyzer instrument (Agilent Technologies, Santa Clara, CA, USA), respectively. Only samples with RIN ≥ 9 were used for subsequent experiments.

### 2.4. Digital PCR

Colorectal cancer patients with both matched blood and serum samples available that had passed quality control were included in the gene expression analysis. Total RNA (150 ng) from blood cells and serum was reverse transcribed in 20 µL reaction volume using a SuperScript™ VILO™ cDNA Synthesis Kit (Thermo Fisher Scientific) according to the manufacturer’s manual. Digital PCR was performed with the QuantStudio™ 3D Digital PCR System (Life Technologies, Carlsbad, CA, USA) according to the manufacturer’s recommendations. Reactions were performed in a final volume of 18 µL containing Quantstudio 3D Digital Master Mix v2 (9 µL), TaqMan assay-FAM (0.6 µL), TaqMan assay-VIC (0.6 µL), nuclease-free water (2.8 µL) and 7.5 ng of template cDNA (5 µL). Samples (16 µL) were loaded onto chips using the QuantStudio 3D Digital Chip Loader (Life Technologies, Carlsbad, CA, USA) and cycled according to the following parameters: 96 °C for 10 min, followed by 20 cycles at 59 °C for 2 min and 98 °C for 30 s, 25 cycles at 57 °C for 2 min and 98 °C for 30 s and a final elongation step at 57 °C for 7 min and then 10 °C hold. All samples were run in duplicate. The TaqMan probes (Life Technologies, Carlsbad, CA, USA) used for target gene detection were *GUCY2C* (Hs00990120_m1), *MMP7* (Hs01042796_m1), *JAG1* (Hs01070032_m1), *PGF* (Hs00182176_m1) and *PTGS2* (Hs00153133_m1). The *MTR* gene (Hs01090026_m1), whose expression was stably shown by qPCR, was selected among five commonly used endogenous genes and employed as housekeeping for expression normalization. After thermo-cycling, the QuantStudio™ 3D Digital PCR Instrument (Life Technologies, Carlsbad, CA, USA) and the QuantStudio™ 3D AnalysisSuite™ Software (Life Technologies, Carlsbad, CA, USA) were used to collect and analyze the end-point fluorescence data of each chip according to the manufacturer’s instructions. The absolute concentration of the target gene per µL of input DNA (copies/µL) was obtained and then normalized using the MTR expression. Finally, the ΔCt was calculated for each sample (ΔCt = no. FAM/no. VIC).

### 2.5. Statistical Analyses

Gene expression levels between the two groups were compared using unpaired Student’s t-tests (Welch’s correction was applied for data groups with unequal variances) or the Mann–Whitney or Wilcoxon matched-pairs tests, as appropriate. Outliers were identified by applying the ROUT test. To establish the cut-off point with the best predictive capacity for each biomarker as well as their combination, the Youden’s index was calculated from the sensitivity and specificity values of each of the coordinates obtained in the ROC curve. Once the cut-off point for each biomarker was established, sensitivity, specificity, positive predictive values (PPV) and negative predictive values (NPV) were calculated, as well as the area under the ROC curve (AUC). A significance level of 0.05 was considered for hypothesis testing. In addition, the relationship between each biomarker and the response to treatment was analyzed using Pearson’s chi-squared test or, in cases in which the applicability conditions were not met, Fisher’s exact test. Survival curves were estimated with the Kaplan–Meier method (95% confidence intervals (CIs)), and the differences between subgroups were compared using the log-rank test. Data were analyzed with IBM SPSS Statistics 19 software and GraphPad Prism 7 (GraphPad Software, San Diego, CA, USA).

## 3. Results

### 3.1. Differential Gene Expression in Metastatic CRC Patients

**Figure 1:**
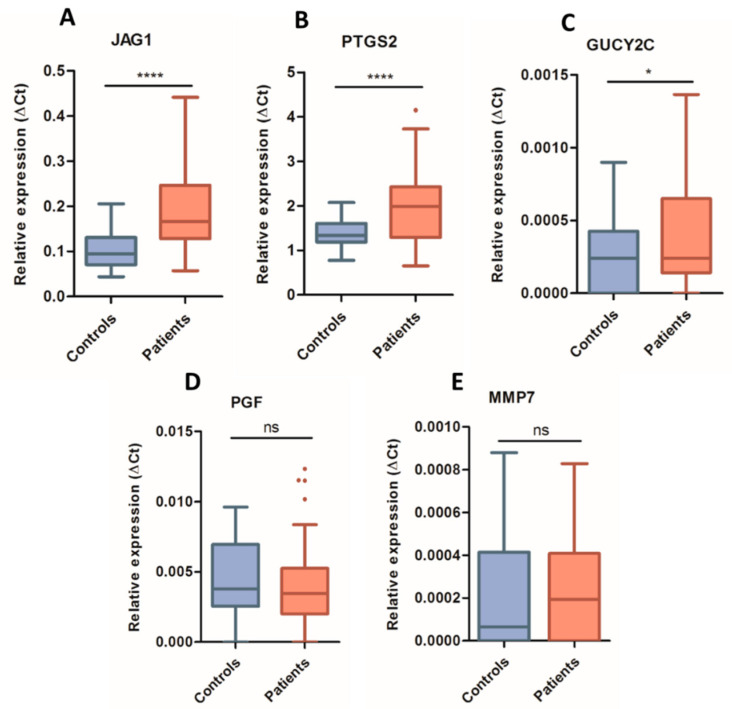
Relative quantification of gene expression levels by digital PCR in whole blood samples. Expression levels of *JAG1* (**A**), *PTGS2* (**B**), *GUCY2C* (**C**), *PGF* (**D**) and *MMP7* (**E**) genes were determined in whole blood samples from metastatic colon cancer patients (red) and healthy controls (blue), normalized by *MTR* expression and calculated by the ΔCt method. In box plots, boxes show the interquartile range (IQR), whiskers indicate Q1-1.5xIQR and Q3 + 1.5xIQR values, inner lines indicate medians. (*) *p* < 0.05; (****) *p* < 0.0001; (ns) not significant.

In total, 59 CRC blood samples were analyzed for gene expression by dPCR. Three of the five candidate angiogenesis-associated biomarkers demonstrated statistically significant differences in expression between CRC patients and healthy controls in the blood samples (Figure 1). A significant upregulation of *JAG1* (*p* < 0.0001), *PTGS2* (*p* < 0.0001) and *GUCY2C* (*p* = 0.0349) genes was observed in blood cells from CRC patients compared to controls (Figure 1A–C). In contrast, *MMP7* and *PGF* presented similar expression levels between both study groups (Figure 1D,E).

**Figure 2:**
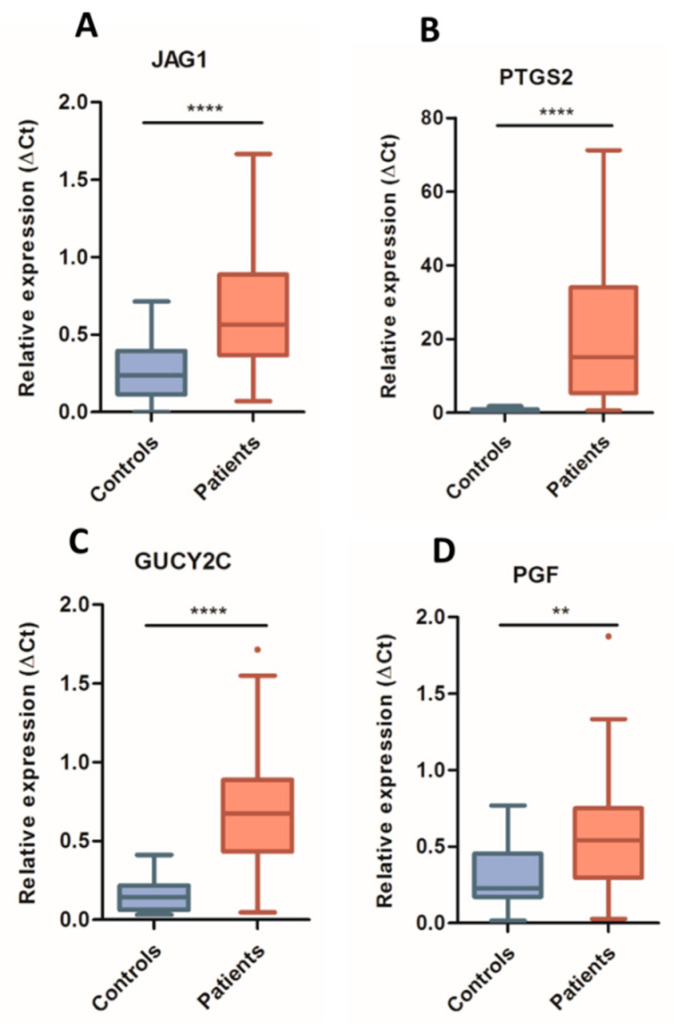
Relative quantification of gene expression levels by digital PCR in serum samples. Expression levels of *JAG1* (**A**), *PTGS2* (**B**), *GUCY2C* (**C**) and *PGF* (**D**) genes were determined in serum from metastatic colon cancer patients (red) and healthy controls (blue), normalized by *MTR* expression and calculated by the ΔCt method. In box plots, boxes show the interquartile range (IQR), whiskers indicate Q1-1.5xIQR and Q3 + 1.5xIQR values, inner lines indicate medians. (**) *p* < 0.01; (****) *p* < 0.0001; (ns) not significant.

Next, we selected the most upregulated genes (*JAG1*, *PTGS2* and *GUCY2C*) in the blood cells of CRC patients to determine the presence of circulating mRNA in the matched serum samples. As shown in Figure 2, the three modulated genes showed a clear and statistically significant (*p* < 0.0001) upregulation in serum from these patients (Figure 2A–C) relative to controls. Furthermore, although *PGF* was not significantly increased in blood cells from CRC patients, it was also analyzed because of its role promoting angiogenesis, in resistance to antiangiogenic therapy and as an alternative pro-angiogenic ligand to VEGF-A in the VEGF pathway. *PGF* showed a clear upregulation (*p* < 0.01) in our cohort of patients (Figure 2D).

### 3.2. Different mRNA Levels in Serum and Whole Blood Samples

**Figure 3:**
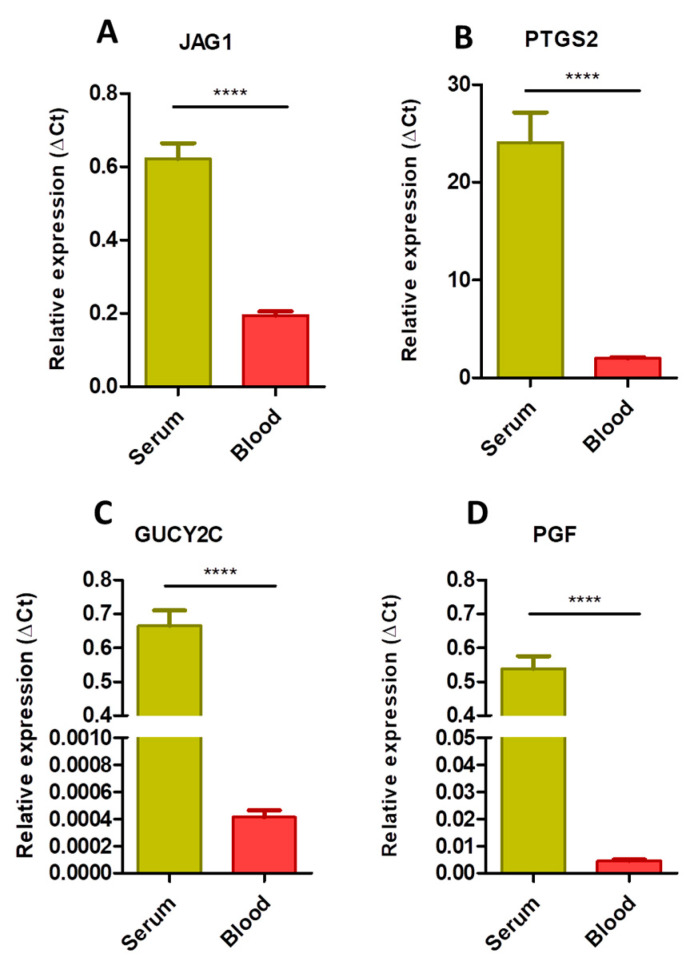
Comparison of gene expression levels obtained by digital PCR in matched serum and blood cell samples from patients with metastatic colon cancer. Expression levels of *JAG1* (**A**), *PTGS2* (**B**), *GUCY2C* (**C**) and *PGF* (**D**) genes were normalized by *MTR* expression and calculated by the ΔCt method (data represent mean ± SEM). (****) *p* < 0.0001.

To analyze the differences regarding the mRNA levels present in both types of samples, the expression of each individual gene obtained by dPCR in both fluids was compared in the CRC patient group. Our results showed that circulating mRNA levels in the serum of all genes were significantly more abundant than in blood cells (*p* < 0.0001; Figure 3), with *GUCY2C* and *PGF* being especially low in the whole blood compared with serum samples (Figure 3C,D). This result reveals that serum is a reliable source of circulating mRNA-based biomarkers.

### 3.3. Correlation Analysis of Candidate Biomarkers

Analysis of the possible correlation between the selected biomarkers, which are related to angiogenesis events, showed a low correlation coefficient. Specifically, *JAG1*, *GUCY2C* and *PTGS2* mRNA levels in serum showed irrelevant or modest correlations with each other (r = 0.26–0.42; *p* < 0.05). On the other hand, only a moderate correlation between *JAG1* and *GUCY2C* expression in blood cells was detected (r = 0.55; *p* < 0.0001). Additionally, we evaluated possible associations for each individual biomarker in serum and blood cells, but no significant correlations were found in our study population suggesting that whole blood and serum can be considered as independent measurement sources.

### 3.4. Sensitivity and Specificity as Biomarker Signatures in Blood and Serum

**Figure 4:**
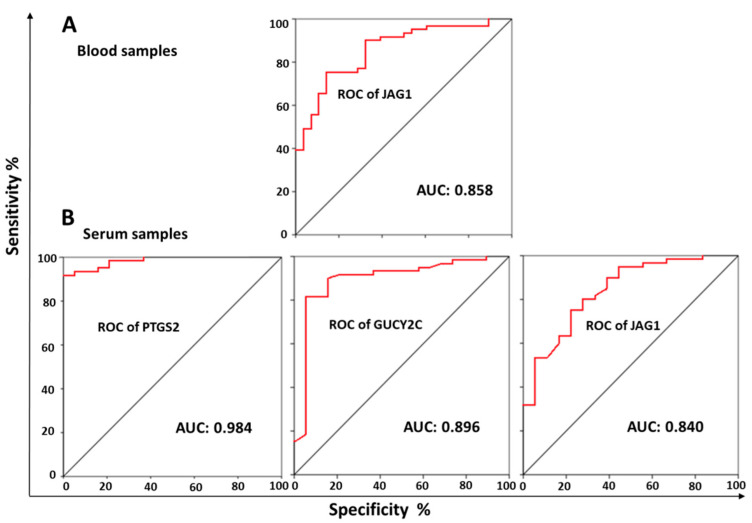
Receiver operating characteristic (ROC) curves analysis. The area under the ROC curve (AUC) with 95% of confidence intervals (CIs) was obtained to evaluate the diagnostic accuracy of individual blood-derived circulating mRNAs of *JAG1* (**A**) and individual serum-derived circulating mRNAs of *PTGS2*, *GUCY2C* and *JAG1* (**B**).

Given the high expression of *JAG1*, *GUCY2C*, *PTGS2* and *PGF* genes in CRC, we performed a ROC curve analysis and calculated the AUC to investigate their individual ability to discriminate between CRC patients and healthy subjects. We also combined these genes in different panels to examine their potential diagnostic advantages. Overall, the blood-derived results showed that *JAG1* was the biomarker with the best performance, with an AUC value of 0.858 (95% CI, 0.778–0.937) (Figure 4). Analysis of the other genes did not show a great discriminatory capacity (AUC < 0.80). Moreover, the different combinations of the genes analyzed in blood did not provide additional benefits.

Remarkably, ROC analyses of circulating mRNA in serum showed higher AUC values than in blood cells for almost all individual biomarkers, most notably *PTGS2* with an AUC of 0.984 (95% CI, 0.963–1.000) and *GUCY2C* with an AUC of 0.896 (95% CI, 0.803–0.988). In this type of sample, *JAG1* showed a similar performance to that found in blood (AUC of 0.840, 95% CI, 0.737–0.943).

Regarding serum pairs, the *JAG1*-*GUCY2C* and *JAG1*-*PTGS2* combinations showed an AUC of 0.819 (0.728–0.910) and 0.831 (0.743–0.743), respectively, which did not improve the discriminatory ability of *JAG1* alone. Similarly, the combination of the *PTGS2* gene with others such as *GUCY2C* and *PFG* (*PTGS2*-*GUCY2C* and *PTGS2*-*PFG*) showed an AUC value of 0.879 (0.805–0.953) and 0.852 (0.772–0.933), respectively; thus, this association did not improve the discriminatory ability of the individual biomarkers. Finally, only the use of three biomarkers (*GUCY2C*, *PTGS2* and *PFG*) showed an AUC value higher than 0.8 (0.802; 95% CI, 0.707–0.897) (Table 2).

### 3.5. Correlation Analysis of Biomarkers, Treatment Response and Metastasis

Analysis of biomarkers to determine their correlation with treatment response showed that only the expression of *GUCY2C* in patients’ serum could correlate with therapeutic response using the antiangiogenic agent. In fact, 76.9% of patients with high serum *GUCY2C* expression had a better course of the disease while progression was observed in only 20% of patients with high *GUCY2C* expression (*p* = 0.047). This significant result was also observed for the combination of *GUCY2C*-*PTGS2* in the serum of patients treated with an antiangiogenic agent (*p* = 0.047). In addition, the serum of patients treated without antiangiogenic agents showed a low expression of *GUCY2C*-*PGF*, which was of statistical significance to predict treatment response (*p* = 0.043). Finally, we analyzed the association between biomarkers and the presence of metastasis. Our results demonstrated that patients with metastases in more than one organ have higher expression of *PTGS2* in serum (*p* = 0.024). In addition, patients with exclusive liver metastasis, as well as those with metastasis in a single organ (*p* = 0.029), have higher expression of *PGF* in blood (*p* = 0.02).

### 3.6. Biomarker Correlation with Overall Survival or Progression-Free Survival

In our study population, overall survival rates were 95.3% at 6 months, 75% at 1 year and 31.2% at 3 years. The probabilities of progression-free survival at 6 months, 1 year and 3 years were 77.5%, 69.7% and 64.4%, respectively (Figure S1). However, no biomarker was significant for overall survival or progression-free survival. In addition, our results were not significant for progression-free survival according to degree of differentiation. Only one significant relationship between overall survival and type of metastasis was observed. Concretely, patients with single-organ metastases had greater survival rates (*p* = 0.002) (Figure S1). For both death and disease progression, all biomarker combinations showed reasonable specificity values but low sensitivity. The most remarkable value was the high blood expression of *JAG1* and *PGF*, with sensitivity and specificity values of 60% to predict disease progression.

## 4. Discussion

Early detection of metastatic CRC is essential for prompt initiation of treatment to improve patient prognosis. Unfortunately, no sensitive biomarkers have been determined to detect this stage of the disease. Therefore, research on novel markers for metastatic CRC is a priority. In this study we analyzed different markers known to be related to angiogenesis, an essential process for the development of the metastatic stage of CRC, in blood and serum to determine their diagnostic ability.

A large number of CRC biomarkers have been analyzed, including DNA, RNA, proteins, volatile organic compounds, metabolites and fecal bacteria, although their clinical application depends on the degree of invasiveness of the procedure. In this context, “liquid biopsy” is becoming an essential methodology for the development of new markers [45]. RNA in blood or serum has shown good diagnostic performance and high sensitivity to distinguish different stages of the disease. Recently, RT-qPCR and dPCR of *MACC1* and *S100A4* (metastasis-associated in colon cancer 1 and S100 calcium-binding protein A4, respectively) transcripts in serum have been correlated with diagnosis, progression-free survival and overall survival of ovarian cancer [46]. In CRC, qPCR of serum *HMGA2* oncofetal protein mRNA correlated significantly with its presence in tumors and has been proposed as a novel diagnostic marker for this disease [47]. Previous studies demonstrated that serum *SALL4* mRNA levels in CRC patients correlated significantly with the degree of tumor invasion and differentiation with high sensitivity and specificity (96% and 95%, respectively), according to ROC analysis [48]. Similarly, tetraspanin 8 membrane protein (*TSPAN8*) mRNA in whole blood of CRC patients also showed notable sensitivity (83.6%) and specificity (58.2%) (AUC = 0.751) to differentiate patients from healthy donors [49]. This protein, which promotes angiogenesis among other functions, has been recently proposed as a potential target for CRC radio-immunotherapy [50].

**Figure 5:**
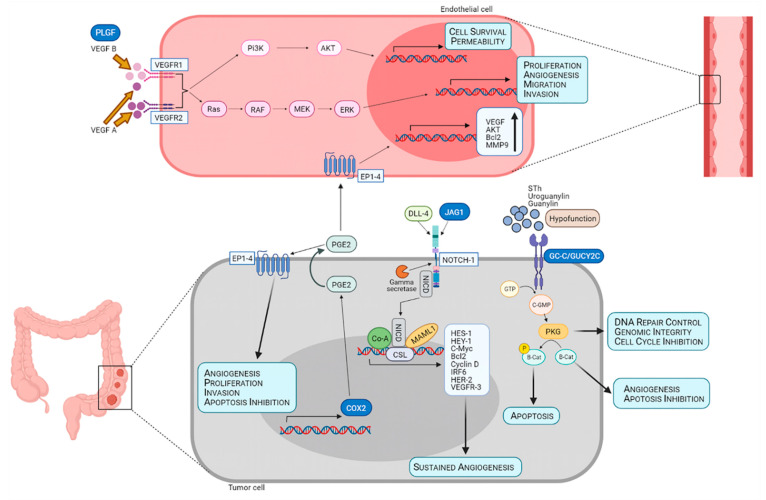
Schematic illustration summarizing the role of JAG1, PTGS2, GUCY2C and PLGF in tumor angiogenesis. PLGF (also termed PGF) may promote angiogenesis in vascular endothelial cells by binding to the VEGF receptor (VEGFR1), thereby increasing the availability of VEGF A for activation of VEGFR2, which exhibits higher kinase activity to induce angiogenesis-promoting signaling and other processes that favor tumor growth. The Notch pathway is activated through the binding of JAG1, among other ligands, to the receptor on the tumor cell surface, releasing the intracellular domain from the membrane that translocates to the nucleus, where it forms a complex to induce the transcription of several target genes that favor sustained angiogenesis. Transcription of *PTGS2* (also termed COX2) mediates the production of prostaglandin E2 (PGE2) by tumor cells, which may act in a paracrine way by binding to prostaglandin receptors (EP1, EP2, EP3 or EP4) on the endothelial cell and inducing proliferation and angiogenesis, or in an autocrine way to further support angiogenesis and tumor promotion. In colon cancer, the tumor-suppressor gene *GUCY2C* (also referred to as GC-C) is upregulated but maintained in a hypoactivated state due to a lack of ligands, resulting in impaired signaling and leading to loss of genomic integrity, apoptosis inhibition and angiogenesis.

Given the complex map of genetic disturbances that may underlie angiogenic mechanisms, it appears necessary to use a wide and heterogeneous group of potential biomarkers, representative of the angiogenic process, key in metastasis and tumor progression. In this context, angiogenesis-related genes were used to analyze their discriminatory power to distinguish between patients with CRC and healthy donors. These genes showed better performance in serum than in blood. In fact, three genes associated with angiogenesis (*JAG1*, *PTGS2* and *GUCY2C*) showed statistically significant differential expression in blood cells of CRC patients compared to controls. Interestingly, serum mRNA levels of *JAG1*, *PTGS2* and *GUCY2C* showed greater differences between CRC patients and healthy controls than those detected in blood. Furthermore, a positive regulation of *PGF* in the serum of CRC patients was detected (but not in blood). Our results support the hypothesis of the overactivation of angiogenesis in metastatic CRC disease through several positively regulated pro-angiogenic factors (Figure 5) that can be detected as circulating mRNA [51].

Analysis of the predictive ability of our selected biomarkers suggested that these pro-angiogenic factors could be clinically relevant in metastatic CRC. Although the presence of *JAG1* in blood showed some discriminatory capacity (AUC of 0.858), the best performance was obtained with the use of serum samples in which *PTGS2*, *GUCY2C* and *JAG1* showed remarkable values, particularly *PTGS2* (AUC of 0.984 (0.963–1.000)) and *GUCY2C* (AUC of 0.896 (0.803–0.988)). To our knowledge, *PTGS2* was detected in CRC tumor tissue and correlated with increased mortality [52]. In fact, recently, *PTGS2* overexpression in tumor tissues of CRC patients (immunohistochemical and qPCR method) was closely associated with clinico-pathological data demonstrating a more pronounced expression in males vs. females [53]. On the other hand, in a large prospective multicenter blinded study conducted by Waldman et al. (2009) [54] that included 257 patients, *GUCY2C* mRNA allowed for the detection of metastases in patients considered stage II. Recently, *GUCY2C* mRNA expression profiling in tissue helped to stage CRC primary tumors and detect occult metastases [55]. In addition, *JAG1* mRNA was the only biomarker that displayed similar notable results in both blood (AUC of 0.858) and serum (AUC of 0.840) samples from CRC patients. This interesting marker, which was already known to be closely related to vasculogenesis regulation, exerts pro-oncogenic functions [56]. In fact, anti-JAG1 antibodies have been proposed as an advanced therapy for cancer patients with high JAG1 tissue expression [57]. In a cohort of 158 CRC patients and using immunohistochemical detection, Sugiyama et al. (2016) [41] demonstrated that high JAG1 expression levels were associated with poor prognosis through promotion of epithelial-to-mesenchymal transition and cell proliferation. Moreover, *JAG1* mRNA expression was analyzed in tumor tissues from 20 patients with squamous cell carcinoma (SCC), showing that increased *JAG1* transcription significantly correlated with poor overall survival [58].

The possibility of combining mRNA biomarkers to improve their CRC diagnostic performance has been extensively tested. In fact, seven mRNAs (annexin A3, C-type lectin domain family 4 (member D), lamin B1, proline rich gla, tumor necrosis factor, vanin 1 and interleukin 2 receptor beta) and three mRNAs (TSPAN8, lectin galactoside-binding soluble 4 and collagen type I alpha 2 chain) in blood were used by Rodia et al. (2016) and Marshall et al. (2010) [49,59], respectively, to detect CRC patients. In the latter study, the biomarker panel showed a specificity/sensitivity of 67.16%/92.54%. Interestingly, the recent inclusion of a new gene (antigen-related cell-adhesion molecule 6, *CEACAM*) in this biomarker panel improved performance, obtaining an AUC of 0.88 (sensitivity, 75%; specificity, 87%) in normal and high-risk/CRC subjects, and an AUC of 0.91 (sensitivity, 79%; specificity, 94%) in normal and low-risk/CCR subjects [60]. Surprisingly, the different combinations of our biomarkers in both serum and blood did not provide additional benefits. The lack of correlation between each individual biomarker in serum and blood cells in our study population supports the idea that whole blood and serum can be considered independent measurement sources. Furthermore, the negative result of combining biomarkers to improve their predictive value supports the recent idea of configuring panels consisting of uncorrelated biomarkers to reveal maximum information about patients [61].

Finally, the study of the relationship between biomarkers and clinical parameters in patients with CRC showed that only *JAG1*-*PGF* in blood reached significant values to predict disease progression. Some in vitro analyses showed that *JAG1* silencing reduced the invasiveness of CRC cells, their growth rate and the expression of some metastasis markers such as MMP-2 and MMP-9 [62]. In contrast, in vivo studies showed that high *JAG1* expression correlated with poor survival in CRC patients [63]. Moreover, increased copy numbers of the *Notch* gene (Jagged-1 ligand) were a negative prognostic factor for survival and were associated with poor prognosis after CRC surgery [41]. Regarding the prediction of treatment response, the expression of the *GUCY2C* gene in serum was the best marker, being useful in predicting patient response both in those treated with antiangiogenic and without antiangiogenic agents. This approach has also been studied in a prospective analysis of first-line therapy with antiangiogenics by Giampieri et al. (2020) [51], who proposed early increase in circulating FGF-2 levels as a biomarker for patients most likely to benefit from this treatment [51]. Previously, also in metastatic colon cancer, increased serum levels of sTRAIL (serum-soluble TNF-related apoptosis-inducing ligand) were proposed as a useful biomarker for early evaluation of patients treated with antiangiogenic agents [64]. Finally, we only observed longer survival in metastatic patients when metastasis was present in a single organ, in accordance with previously studies reporting that patients with a single metastatic site had better prognosis for both cancer-specific survival and overall survival than patients with involvement of multiple organs [65].

## 5. Conclusions

In summary, we analyzed blood and serum samples to detect mRNA of genes involved in the angiogenic process with the aim of finding new biomarkers that may improve the diagnosis and prognosis of metastatic CRC patients. A prospective analysis of peripheral blood samples from 59 metastatic CRC patients showed that dPCR detection of *PTGS2*, *GUCY2C* and *JAG1* upregulation in serum correlated with high discrimination ability. The highest discrimination performance was obtained with the use of *PTGS2* mRNA (AUC of 0.984). In contrast, all biomarker combinations did not significantly improve this discriminatory ability. Interestingly, *GUCY2C* and *GUCY2C*/*PTGS2* serum expression significantly correlated with therapeutic response. However, none of the biomarkers correlated with overall survival or progression-free survival. Although further studies, including studies on non-metastasic and chronic inflammation patients, will be necessary to elucidate their role, these findings suggest that angiogenesis-related genes can be used as potential non-invasive biomarkers for the diagnosis and/or prognosis of patients with metastatic CRC.

## Figures and Tables

**Table 1:** Characteristics of metastatic CRC patients.

Characteristic	CRC Patients
Age (years ± SD)	60.12 ± 11.16
Sex	
*Male*	33
*Female*	26
*RAS* gene	
*Non-mutated*	28
*Mutated*	31
Metastasis	
*One organ*	24
*More than one organ*	35
Therapy	
*Chemotherapy +* *antiagiogenic*	35
*Chemotherapy*	24
Tumor location	
*Rectum*	21
*Transverse/Left colon*	27
*Right colon*	11
Metastatic site	
*Liver*	36
*Lung*	9
*Peritoneum*	7
*Lymph nodes*	5
*Other*	2
Treatment	
*Antiangiogenic treatment*	35
*No antiangiogenic treatment*	24

**Table 2:** ROC parameters for diagnosis of CCR patients with metastasis using serum biomarker combinations.

	AUC	95% CI	PPV	NPV	Sensitivity (%)	Specificity (%)
*JAG1*-*GUCY2*	0.819	0.728–0.910	100	46	63.8	100
*JAG1*-*PTGS2*	0.831	0.743–0.743	100	47.4	66.1	100
*GUCY2*-*PTGS*	0.879	0.805–0.953	100	57.6	75.9	100
*PFG*-*PTGS2*	0.852	0.772–0.933	100	51.4	70.5	100
*GUCY2*-*PFG*- *PTGS2*	0.802	0.707–0.897	100	45.2	60.3	100

AUC, area under the curve; PPV, positive; NPV, negative predictive values.

## Data Availability

Not applicable.

## Supplementary Materials

The following are available online at https://www.mdpi.com/article/10.3390/jcm10112248/s1, Figure S1: Survival of metastatic CRC patients.
