# Supplementary material for: Circulating PTGS2, JAG1, GUCY2C and PGF mRNA in Peripheral Blood and Serum as Potential Biomarkers for Patients with Metastatic Colon Cancer
Source: J Clin Med. 2021 May 22;10(11):2248. doi: 10.3390/jcm10112248 (PMC8196898; doi:10.3390/jcm10112248)
Supplement: Supplementary file 1 [file jcm-10-02248-s001.zip › jcm-1205822-supplementary.pdf]

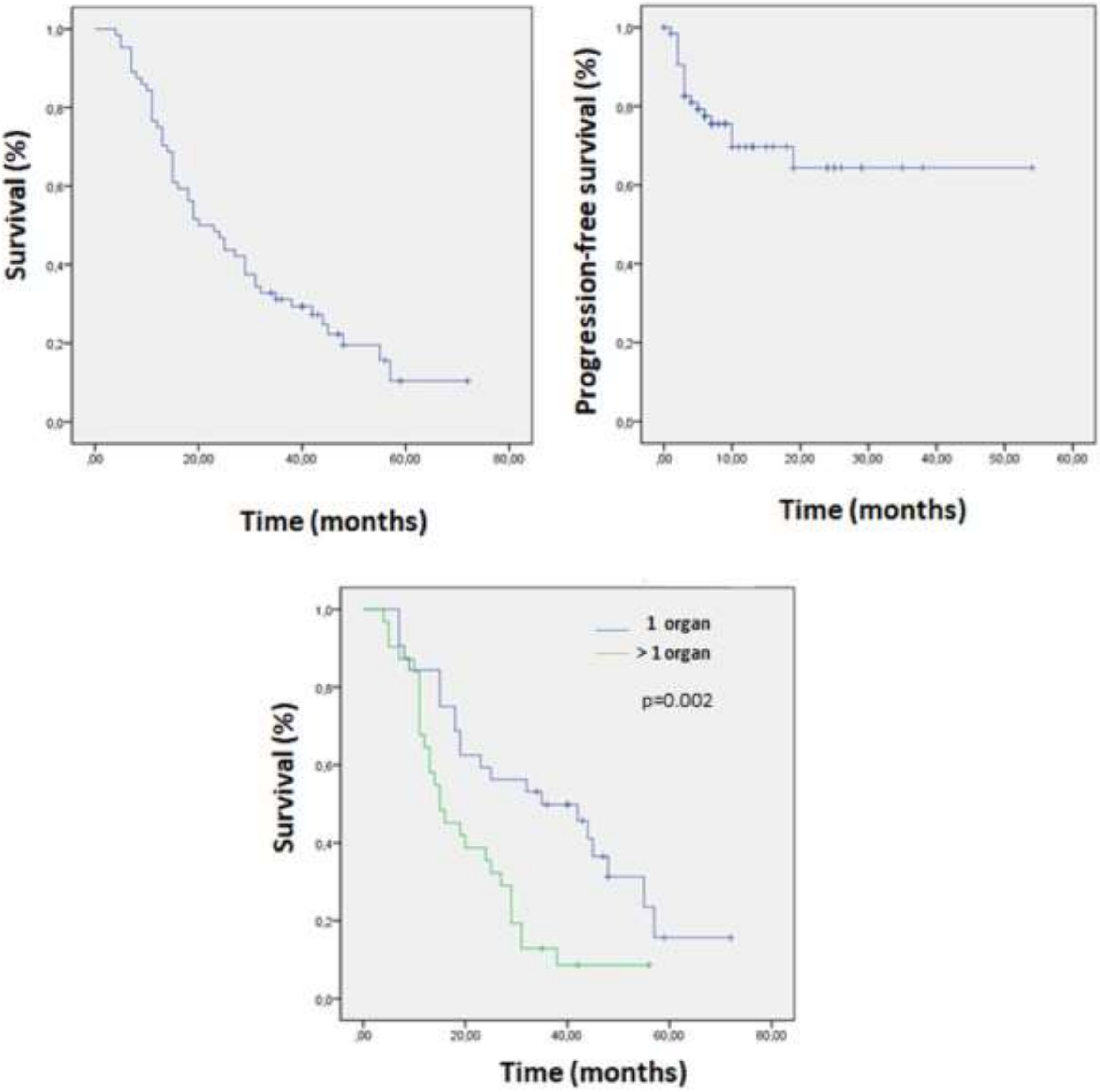

Figure S1. Survival of metastatic CRC patients. General overall survival (A), progression-free survival (B) and overall survival according to type of metastasis (C).
